# Searching for potential *Culicoides* vectors of four orbiviruses in Yunnan Province, China

**DOI:** 10.1186/s13071-025-06679-1

**Published:** 2025-02-24

**Authors:** Zhan Hong Li, Yi Nan Wang, Jia Ming Deng, Le Li, Lian Jiang Yang, Xinq Qiang Chen, Wen Hua Wang, Fu You Lu, Zhong Jie Tang, Dong Mei Wang, Ying Liang Duan

**Affiliations:** 1 Yunnan Tropical and Subtropical Animal Virus Diseases Laboratory, Yunnan Animal Science and Veterinary Institute, Kunming, Yunnan China; 2 Key Laboratory of Transboundary Animal Diseases Prevention and Control (Co-Construction by Ministry and Province), Ministry of Agriculture and Rural Affairs, Kunming, China; 3 Center for Animal Disease Control and Prevention, Tengchong, Yunnan China; 4 Animal Health Supervision Institute, Bureau of Agriculture and Rural Affairs, Ruili, Yunnan China; 5 Animal Health Supervision Institute, Bureau of Agriculture and Rural Affairs, Yingjiang, Yunnan China; 6 Aquatic Workstation, Bureau of Agriculture, Rural Affairs and Science and Technology, Yuanyang, Yunnan China; 7 Center for Animal Husbandry Development of Puer, Puer, Yunnan China; 8 Animal Husbandry and Veterinary Workstation, Bureau of Agriculture and Rural Affairs, Menghai, Yunnan China; 9 Center for Animal Disease Control and Prevention, Jiangcheng, Yunnan China

**Keywords:** *Culicoidesshortti*, *Culicoidesjacobsoni*, *Culicoidesorientalis*, *Culicoidesoxystoma*, BTV, PALV, TIBOV, Vector, China

## Abstract

**Background:**

Some species of *Culicoides* (Diptera, Ceratopogonidae) are major vectors for arboviruses, and Yunnan Province is a key area for arbovirus prevalence in China. Therefore, this study attempts to search for potential *Culicoides* vectors for the common orbiviruses bluetongue virus (BTV), epizootic hemorrhagic disease virus (EHDV), Palyam virus (PALV) and Tibet orbivirus (TIBOV) in Yunnan Province, China.

**Methods:**

*Culicoides* specimens were collected from 16 counties in Yunnan Province, China, using UV traps and tested for BTV, EHDV, PALV and TIBOV through one-step reverse transcription-quantitative polymerase chain reaction (RT-qPCR). A total of 543 conspecific pools of *Culicoides* containing 9895 specimens were tested.

**Results:**

A total of 46 species belonging to 8 subgenera and 2 groups were recognized. A total of 19 species and a *Culicoides* subgenus *Trithecoides* complex were tested using RT-qPCR. One pool of *Culicoides shortti* Smith & Swaminath and one pool of *Culicoides orientalis* Macfie tested positive for BTV, one pool of *Culicoides oxystoma* Kieffer tested positive for PALV, and four pools of *Culicoides jacobsoni* Macfie tested positive for TIBOV. All the tested samples were negative for EHDV, and all the tested *C.* subgenus *Trithecoides* midges were negative for any virus.

**Conclusions:**

*Culicoides shortti* was identified as a potential BTV vector for the first time. *Culicoides jacobsoni* was confirmed as a potential TIBOV vector and *C. orientalis* as a potential BTV vector. *Culicoides oxystoma* was also shown to be a natural carrier of PALV using the RT-qPCR method.

**Graphical Abstract:**

**Figure:**
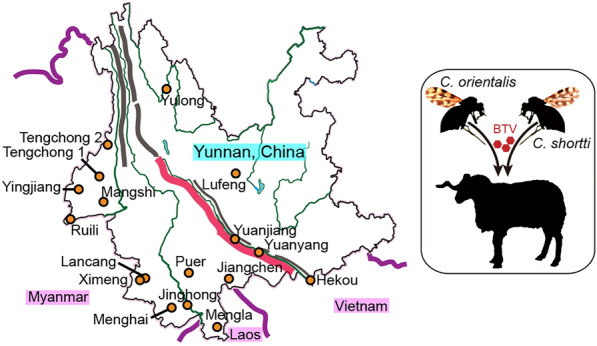

**Supplementary Information:**

The online version contains supplementary material available at 10.1186/s13071-025-06679-1.

## Background

*Culicoides* (Diptera, Ceratopogonidae) is a category of small biting midges measuring 1–3 mm in length [1]. So far, at least 1347 species of *Culicoides* have been recognized and placed into 33 subgenera and 38 species groups [2], and > 300 species have been recorded in China [1]. Some species of *Culicoides* are recognized or potential vectors for > 50 arboviruses belonging to the families *Bunyaviridae*, *Sedoreoviridae* and *Rhabdoviridae* [3].

Among the *Culicoides*-associated viruses, the most concerning are the economically important arboviruses that severely impact farmed mammals and birds. These include bluetongue virus (BTV), epizootic hemorrhagic disease virus (EHDV), Ibaraki virus, Akabane virus (AKAV), African horse sickness virus (AHSV), Chuzan virus (CHUV) of Palyam virus (PALV), equine encephalitis virus (EEV) and so on [4–10]. Among these viruses, BTV, EHDV, PALV, Yunnan orbivirus (YUOV) and TIBOV are familiar orbiviruses in Yunnan, China. BTV and EHDV are *Culicoides*-borne arboviruses, PALV and TIBOV are both mosquito-borne and *Culicoides*-borne viruses, and YUOV is a mosquito-borne arbovirus [11–14].

BTV is known to infect ruminants such as sheep, cattle and goats, typically causing asymptomatic and subclinical symptomatic infections, but it sometimes causes severe diseases in sheep, followed by cattle [4, 15]. Dogs on farms can also occasionally be infected by BTV [16]. BTV was first identified as the pathogen causing bluetongue disease (BT) in sheep in South Africa in the early twentieth century [17]. Subsequently, quite a few outbreaks of BT occurred in Europe and then Asia, resulting in significant livestock losses [5, 18–20]. BTV has been prevalent in both tropical and temperate zones worldwide for a long time. Recently, an outbreak of BT occurred among cattle in Tunisia in 2020 [21], and in 2022, an outbreak with 7% mortality affected 300 sheep in Iran [22]. Additionally, two major BT epidemics in sheep spread rapidly in France and The Netherlands in 2023, causing significant economic losses [23–25].

EHDV is the pathogen of epizootic hemorrhagic disease (EHD), first recognized as a highly fatal disease in wild white-tailed deer in America in 1955 [26]. Similar to BTV, EHDV mainly infects ruminants such as deer, bovines and goats, with deer followed by cattle being the main victims of EHD [4, 7]. EHD has caused mass deaths of deer in North America [27] and cattle in Japan [28, 29]. Ibaraki virus, which belongs to EHDV-2, was the primary cause of EHD in Asia [7, 28, 29].

To date, PALV consists of 13 serological groups: Palyam, Kasba (Chuzan), Vellore, Abadina, D’Aguilar, Nyabira, CSIRO Village, Marrakai, Gweru, Bunyip Creek, Petero, Marondera and Kindia [30]. Unlike BTV and EHDV, these serological groups are named after locations. CHUV, which causes bovine congenital disease, was first recognized in Japan and is prevalent in Asia [7, 31]. However, it was later classified under the Kasba serotype [32].

TIBOV was first isolated from mosquitos in Tibet, China [12], and subsequently isolated from *Culicoides* and mosquitos in China [13, 33–35] and Japan [36]. TIBOV infection in cattle and goats was detected through serological tests [37], suggesting TIBOV as an arbovirus. So far, none of the severe cases have been associated with TIBOV, but it remains uncertain whether a TIBOV-associated disease will occur in the future.

Yunnan Province is located in southwest China within the tropical and subtropical zones (between approximately 21.13 and 29.25°N). It is a major area for arbovirus epidemics in China and borders Myanmar, Laos and Vietnam. The prevalence of BTV and EHDV in Yunnan has been extensively investigated [38–41] since the first outbreak of bluetongue disease in 1979 [20] and the first case of suspected Ibaraki disease reported in 1985 [42] on the Chinese mainland. Some TIBOV and PALV strains have also recently been isolated in Yunnan Province [13, 35, 37, 43–45]. The prevalence of such viruses prompts us to search for more potential vectors of these viruses in Yunnan Province, China.

## Methods

### Samples

**Fig. 1:**
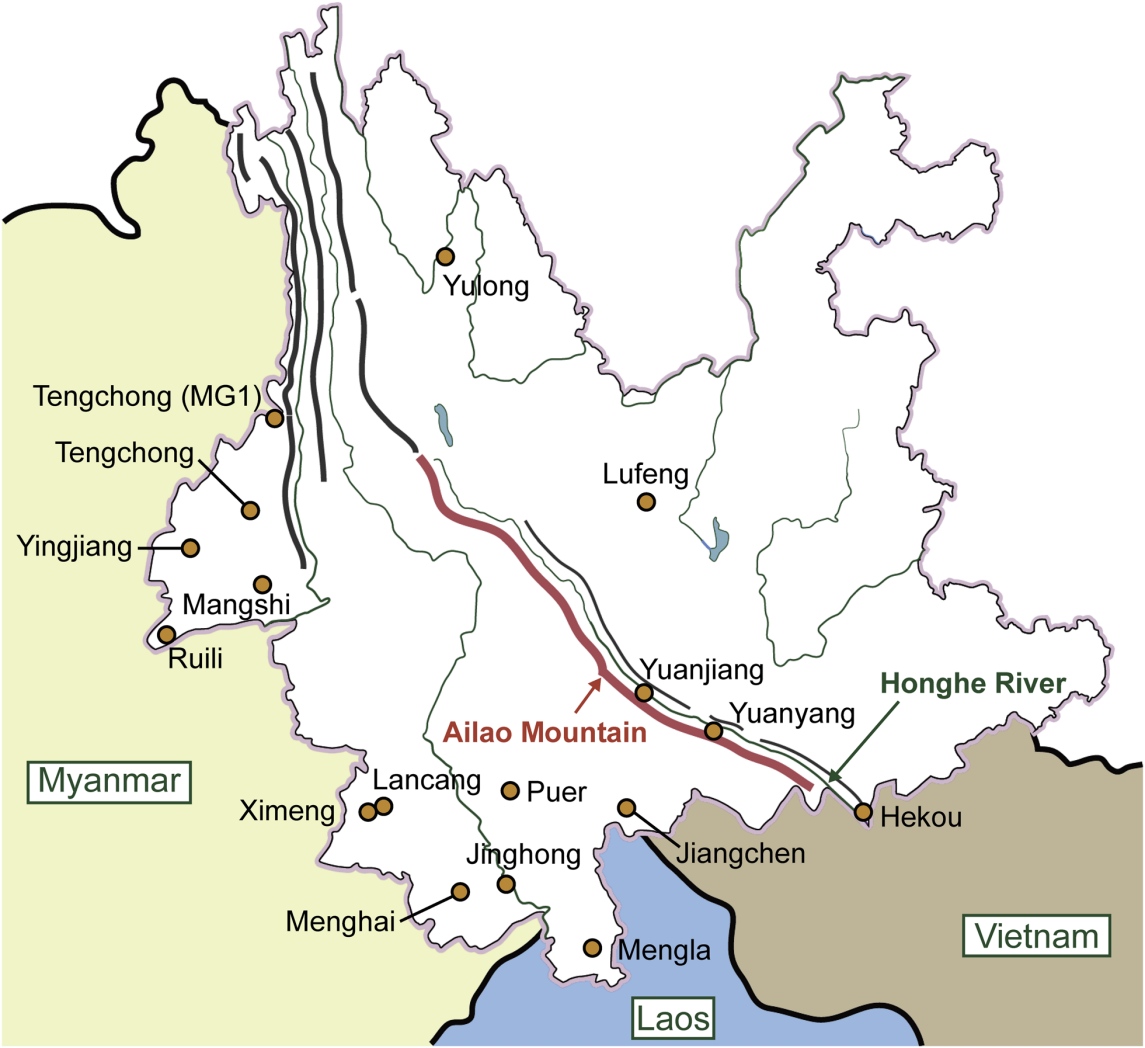
Sketch map for collection sites and Yunnan Province, China. The map of Yunnan Province, China, with major rivers (green color) came from a standard Yunnan map, and the major mountains were hand-painted and shown as thick lines. Probable positions of the counties were marked by circles, and associated collection sites closely surrounded them. MG1 represented the place for collections of MG1 and TCSx in Tengchong County

Biting midges collected between 2021 and 2024 on 23 farms and 1 elephant rescue station across 16 counties in Yunnan Province, China, were used in this study (Fig. 1, Table 1). Midges were lured using battery-powered UV-light traps (Yaoyu Electronics Co., Ltd., Zhangzhou, China) and collected in bottles containing 75% ethanol. The traps were hung in livestock pens as close to the animals as possible while maintaining a proper distance to prevent them from being touched by the livestock. The UV-light traps ran from approximately 4—6 p.m. (before sunset) to 9—10 a.m. (after sunrise) the following day. Subsequently, collected biting midges were transferred to tubes containing 75% ethanol at room temperature and stored at 4 °C in the laboratory until use.

**Table 1:** Collection sites and collections used in this study

County	Collection ID	Penned animal	Collection date	Exact coordinates		
				Latitude (°N)	Longitude (°E)	Elevation (m)
Hekou	O1	Cattle	14-Aug-2022	22.533	103.981	84
Hekou	O2	Goat	14-Aug-2022	22.598	103.931	222
Jiangcheng	M1	Cattle	12-Aug-2022	22.630	101.861	1187
Jiangcheng	M2	Cattle	12-Aug-2022	22.618	101.888	1092
Jinghong	EV21	Elephants	16-Jun-2021	22.179	100.856	737
Lancang	V1	Cattle	27-Aug-2022	22.762	99.763	1877
Lufeng	J1	Cattle	6-Aug-2022	25.004	101.908	1423
Mangshi	MS	Cattle	13-Sep-2023	24.341	98.484	897
Menghai	X1	Chicken	29-Aug-2022	21.994	100.436	1131
Menghai	X2	Cattle and Sheep	29-Aug-2022	22.044	100.482	1176
Mengla	MLa21	Cattle	17-Jun-2021	21.556	101.609	643
Puer	T2	Cow	25-Aug-2022	22.779	100.910	1189
Ruili	R1	Sheep	19-Aug-2022	23.969	97.736	836
Tengchong	MG1^**a**^	Sheep	20-Sep-2024	25.717	98.545	2004
Tengchong	TCSx^**a**^	Sheep	15-Jun-2024	NA	NA	NA
Tengchong	Q1	Buffalo	18-Aug-2022	25.215	98.488	1818
Tengchong	TC-1	Cattle	16-Sep-2023	25.062	98.542	1624
Ximeng	W1	Cattle	28-Aug-2022	22.651	99.640	1015
Yingjiang	S1	Cattle	20-Aug-2022	24.728	97.881	867
Yingjiang	S2	Goat	20-Aug-2022	24.710	97.960	781
Yuanjiang	N1	Cattle	13-Aug-2022	23.568	102.011	411
Yuanyang	K1	Goat	10-Aug-2022	23.200	102.893	215
Yuanyang	K2	Cattle	10-Aug-2022	23.206	102.890	244
Yulong	G1	Cattle	3-Aug-2022	27.036	100.069	1825

^**a**^MG1 and TCSx are located in the same town, but we did not acquire the coordinates of TCSx

### Sorting *Culicoides*

*Culicoides* were identified using the morphological keys of Wirth [46] and Yu et al. [1], and specimens from the dominant species in each collection were chosen for viral detection. Typically, the parous/gravid females without a blood meal were used for viral detection. Since no parous/gravid females are observed in the *C.* subgenus *Trithecoides*, nulliparous-like females without a blood meal from these species were also used. Additionally, a few blood-fed females were used when the parous females of the species of interest were rare.

### Digesting midges

A non-destructive digestion method was used to extract the total nucleic acids from pools of specimens [47]. Briefly, sorted specimens were placed into PCR tubes containing 60 μl tissue lysis buffer (TIANGEN, Tiangen, Beijing, China) with 0.2 mg/ml proteinase K (TIANGEN) and incubated at 30 °C for 16 h. Each tube contained a pool of conspecific midges with the same status (parous/gravid, nulliparous, or blood fed) and from the same collection. In principle, every pool was comprised of ≤ 20 midges, with ≤ 15 midges large in size. Because the positive rate of BTV in *Culicoides* was previously estimated to be ≤ 5% (= 1/20) using RT-qPCR tests on individual midges [38], the size of the pool was controlled at approximately 20 midges per pool in this study to minimize the likelihood of loading two positive midges in one pool.

### Reverse transcription‑quantitative PCR

The samples were tested using two rounds of reverse transcription‑quantitative PCR (RT-qPCR) for BTV, EHDV, PALV and TIBOV. The primers and probes against BTV [48], EHDV [49] and TIBOV [50] were used according to previous reports, while the primers and probes against PALV were newly designed based on Chinese CHUV strains and closed PALV strains (Table S1). For each reaction, a 2-μl aliquot of nucleic acid sample was added to 20 μl of reaction solution prepared using a PrimeScript RT-qPCR Kit (#RR600A, Takara), according to the manufacturer’s instructions. Then, 2 μl of positive control (viral DNA) and 2 μl of negative control (distilled water) were added to assess the validity of RT-qPCR. The RT-qPCR was performed on a Fast7500 Realtime PCR machine (ABI) at the following cycling conditions: 42 °C, 5 min; 95 °C, 10 s; 95 °C for 10 s, 60 °C for 34 s, 40 cycles. Fluorescence was measured at the end of each extension step. For the comparability of the Cq values from different batches of RT-qPCR, the threshold was manually set as 0.015, which was above all the noise signals.

**Fig. 2:**
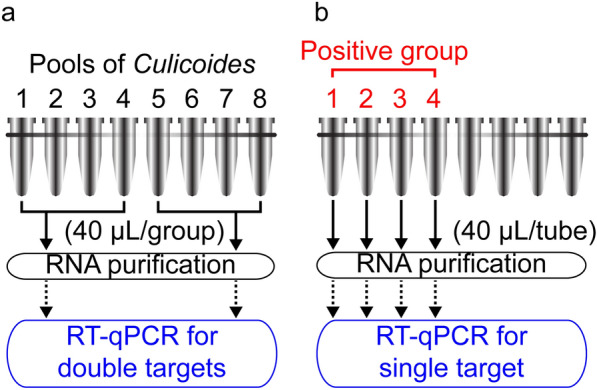
Diagrammatic sketch of the RT-qPCR process. **a** Primary detections, **b** further detections

For primary screening, groups of lysates were submitted for detection using dual-channel RT-qPCR targeting BTV & EHDV and PALV & TIBOV, respectively (Fig. 2a). Briefly, every 40 μl of lysate combined by four pools of lysate (10 μl per pool) was submitted to purify the nucleic acids, using the MagMAX^™^-96 Viral RNA Isolation kit (Ambion, Thermo Fisher Scientific, Waltham, MA, USA) and MagMAX^™^ Express-24 machine (Ambion), and a 2 μl aliquot of eluted nucleic acid was detected using the RT-qPCR.

If only one group was positive for any virus during the primary screening, the four pools of the group were submitted for further detection one by one (Fig. 2b). Briefly, a 40 μl aliquot of lysate from the chosen pool was submitted to purification as described above, and 2 μl of eluted nucleic acid was detected using single-channel RT-qPCR for each virus.

Due to the high cost of the MagMAX^™^-96 Viral RNA Isolation kit, two rounds of RNA purification were used to reduce the cost of reagents and plates (Fig. 2). For example, 96 RNA purification reactions are typically required for 96 pools, but our process required a total of 24 + 4 × N (N = number of positive groups) reactions for 96 pools. If N < 18, our strategy is more economical, and a positive rate of ≥ 75% (18 positive groups/24 groups) is rare.

### Mounting specimens

For the pools positive for a virus, the species and status of the *Culicoides* in these pools were reconfirmed morphologically.

The representative specimens from positive pools were mounted and photographed. Briefly, digested specimens were washed with distilled water twice in PCR tubes and dehydrated using 75% ethanol for 10 min, 85% ethanol for 10 min and 100% ethanol for 3—5 h. Subsequently, the specimens were incubated in a 1:1 (v/v) ethanol-clove oil mixture for 1 day and then incubated in 100% clove oil for at least 24 h. Finally, each prepared *Culicoides* specimen was cut into four parts, i.e. the head, thorax, wing and abdomen, using a tenuous needle and mounted on a slide using a neutral balsam (#E675007, BBI Co., Ltd., Shanghai, China) and four small cover glasses, respectively. Mounted specimens were air dried for several days and kept at room temperature.

### RT-PCR and electrophoresis

One-step RT-PCR was used to amplify the viral fragments for the samples with Cq ≤ 30. Briefly, 5 μl of nucleic acid was added to 20 μl of reaction solution confected using a PrimeScript^™^ One Step RT-PCR Kit Ver.2 (Takara) and specific primer pairs for BTV, PALV and TIBOV, respectively (Table S1), according to the manufacturer’s instruction. The primers for BTV were cited from a previous report [47]. The primers for PALV were designed by us and matched the Chinese CHUV strains SZ187 (NCBI: KT002594.1) and GX871 (NCBI: KT887186.1), as well as closed PALV strains, while the primers for TIBOV were designed by us based on public TIBOV strains. Additionally, 5 μl of water was added to the reaction solution as a negative control (NC). The reaction was performed on a ProFlex PCR System (ABI, Foster City, CA, USA) at the following cycling conditions: 50 °C for 30 min, 95 °C for 3 min and 95 °C for 30 s, annealing at 55 °C for 30 s, and extension at 72 °C for 70 s for 35 cycles.

A 5 μl aliquot of each PCR product was added into a groove of 1.2% agarose gel containing Goldview I dye (Gentihold, Beijing, China). The DNA samples were separated using electrophoresis (120 V, 35 min), and fluorescent bands were screened using a Gel Doc^™^ XR + System with Image Lab™ software (Bio-Rad, Hercules, CA, USA). The exposure time was automatically determined by the light intensity of strong bands.

## Results

### *Culicoides* species

**Fig. 3:**
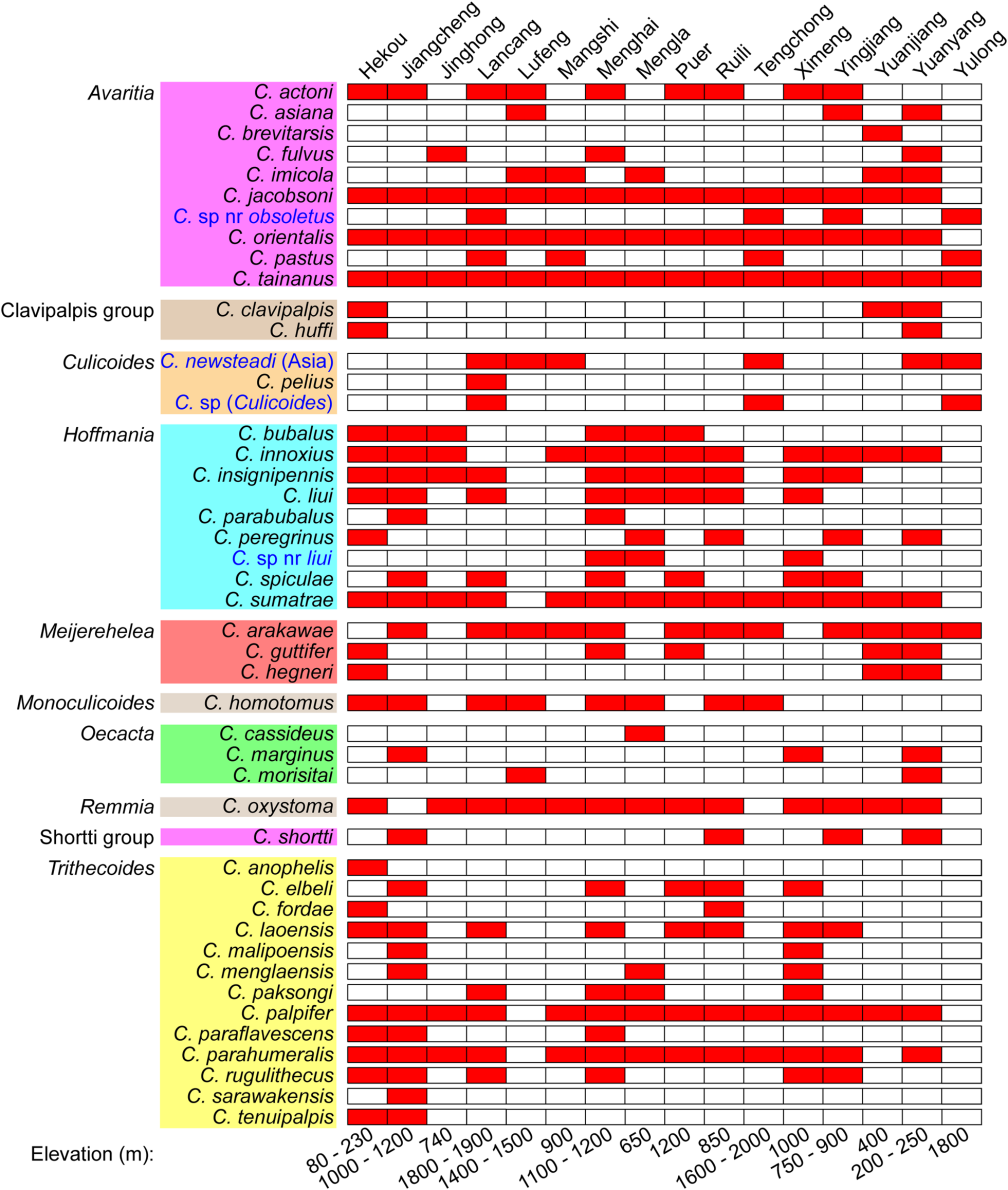
*Culicoides* spp. and their distributions. A total of 46 recognized *Culicoides* spp. belonging to 8 subgenera and 2 groups found in the collections used in this study are listed on the left. All the counties and the altitude range of associated collection sites are shown. Red indicates the distribution of *Culicoides* spp

A total of 46 species containing two new species were found in the collections collected from 24 sites (Table 1) from 16 counties of Yunnan Province, China. The two new species were marked as *Culicoides* sp. (*Culicoides*) and *Culicoides* sp. near *liui* Wirth & Hubert, respectively. A list of all the species and their distributions is shown in Fig. 3, and dominant species were counted (Table S2). These species belonged to eight subgenera (i.e. *Avaritia* Fox, *Culicoides* Latreille, *Hoffmania* Fox, *Meijerehelea* Wirth & Hubert, *Monoculicoides* Khalaf, *Oecacta* Poey, *Remmia* Glukhova and *Trithecoides* Wirth & Hubert) and two groups (i.e. Clavipalpis group and Shortti group). *Culicoides tainanus* Kieffer was the most widespread species, which appeared in all of the 16 counties, followed by *Culicoides jacobsoni* Macfie and *Culicoides orientalis* Macfie, which appeared in 15 counties (Fig. 3).

### RT-qPCR detection

A total of 543 conspecific pools of *Culicoides* containing 9895 specimens were submitted to RT-qPCR detection for BTV, EHDV, PALV and TIBOV (Table S3, Additional file 1). These specimens belonged to 19 species and a *C.* subgenus *Trithecoides* complex (Table S3).

**Fig. 4:**
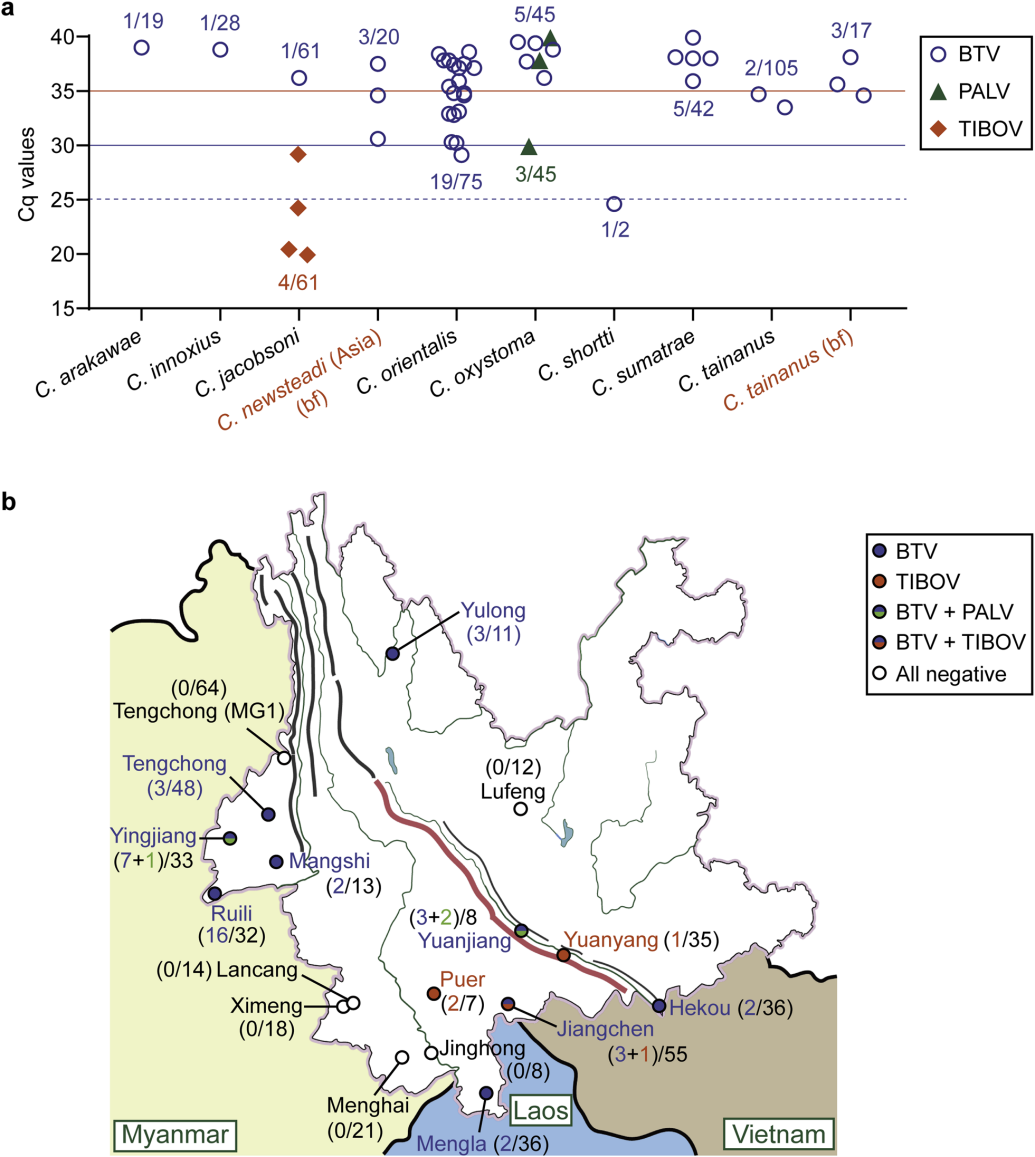
Distributions of the positive midges. **a** All the Cq values of pools positive for BTV, PALV and TIBOV are shown in scatter diagram, horizontal axis displays the categories of pools, and the numbers of positive pools/tested pools are labeled. **b** Viral circulations are marked on the map according to the RT-qPCR results, and the numbers of positive pools/tested pools excluding *Trithecoides* spp. are labeled

The distributions of Cq values in species and the viral circulations in counties were shown, and the numbers of positive pools/tested pools excluding *Trithecoides* spp. were labeled (Fig. 4). For parous/gravid midges, nine pools of *C. orientalis*, one pool of *Culicoides shortti* Smith & Swaminath and two pools of *C. tainanus* were positive for BTV (Cq ≤ 35); one pool of *Culicoides oxystoma* Kieffer was positive for PALV (Cq ≤ 30), while four pools of *C. jacobsoni* were positive for TIBOV (Cq ≤ 30) (Fig. 4a, Table S3). Moreover, 26 pools of conspecific specimens and 2 pools of *C. oxystoma* were faintly positive (Cq > 35) for BTV and PALV, respectively (Table S3). The pools of the *C.* subgenus *Trithecoides*, including pools of conspecific *Culicoides laoensis* Howarth, were all negative for any target (Table S3). No EHDV was detected by the single-channel RT-qPCR, although very weak signals of EHDV were measured using dual-channel (BTV and EHDV) RT-qPCR in a few groups during primary scanning. BTV was widespread especially at the four counties in western Yunnan, and TIBOV was mainly distributed in the south Yunnan (Fig. 4 b).

**Fig. 5:**
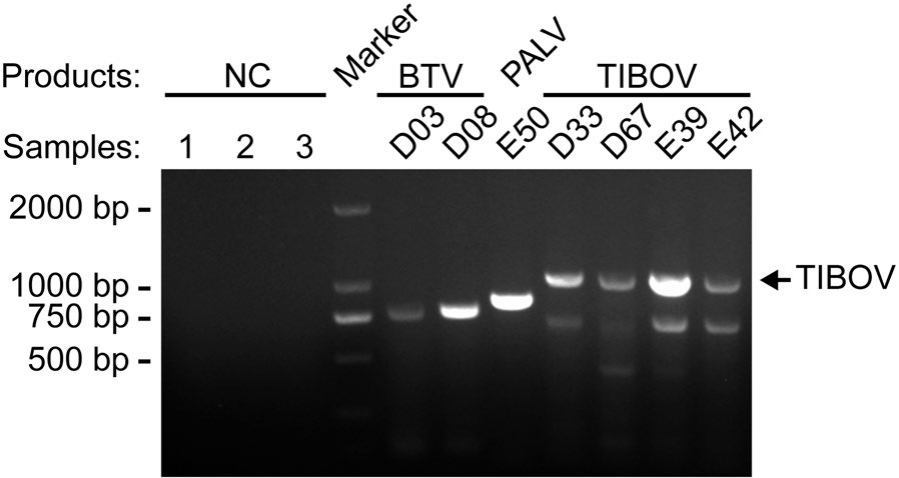
Electrophoresis for RT-PCR products. The target DNA bands of the samples (pools) with Cq ≤ 30 and three negative controls (NC1-3) for BTV, PALV and TIOBV, respectively. Sample (pool) IDs and RT-PCR targets (BTV, PALV and TIBOV) are labeled

Detailed data of the seven pools with Cq values ≤ 30 are shown in Table 2; these results were verified by RT-PCR followed by electrophoresis (Fig. 5).

**Table 2:** The pools of conspecific midges confidently positive (Cq ≤ 30) for BTV, PALV and TIBOV

Species	Pool			Collection			RT-qPCR result	
	ID	Status	Midge^**a**^	County	Farm	Date	Virus	Cq value
*Culicoides jacobsoni*	D33	Parous	20	Yuanyang	Cattle	10-Aug-2022	TIBOV	24.2
*C. jacobsoni*	D67	Parous	20	Jiangcheng	Cattle	12-Aug-2022	TIBOV	29.1
*C. jacobsoni*	E39	Parous	20	Puer	Cow	25-Aug-2022	TIBOV	19.9
*C. jacobsoni*	E42	Parous	17	Puer	Cow	25-Aug-2022	TIBOV	20.4
*C. orientalis*	D03	Parous	21	Ruili	Sheep	19-Aug-2022	BTV	29.1
*C. oxystoma*	E50	Parous	20	Yingjiang	Cattle	20-Aug-2022	PALV	29.9
*C. shortti*	D08	Parous	22	Ruili	Sheep	19-Aug-2022	BTV	24.6

^**a**^Number of conspecific midges in a pool

**Fig. 6:**
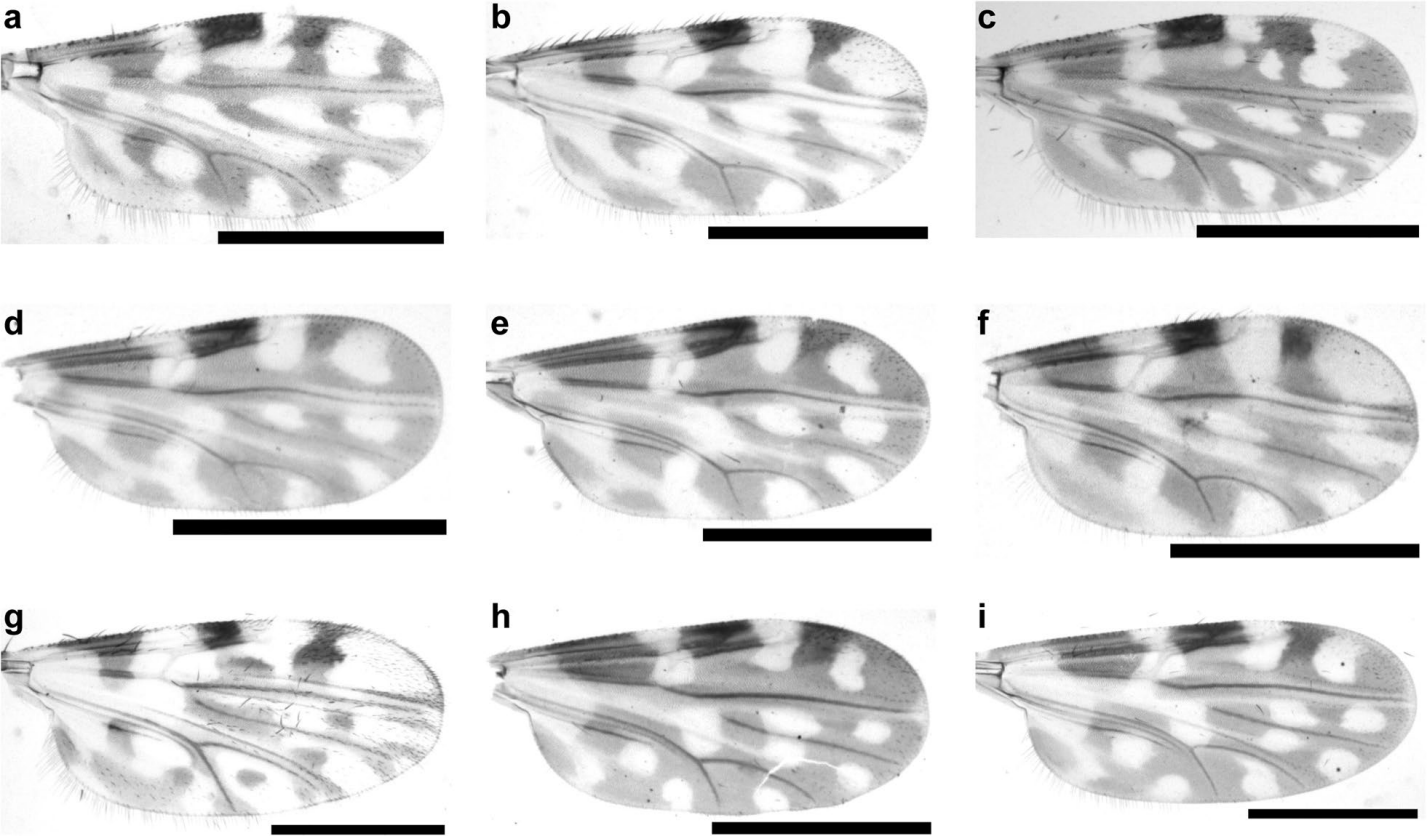
Photos of the wing patterns of representative specimens. The species and pool IDs are as follows: **a** *Culicoides shortti* (D08), **b** *C. orientalis* (D03), **c** *C. oxystoma* (E50), **d** *C. jacobsoni* (D33), **e** *C. jacobsoni* (E39), **f** *C. tainanus* (G24), **g** *C. newsteadi* (Asia) (C16), **h** *C. sumatrae* (G42) and **i** *C. innoxius* (A04). Scale bar = 0.5 mm

The wing patterns of representative specimens from positive pools are shown in Fig. 6.

## Discussion

Some species of *Culicoides* are vectors of arboviruses that are significant for domesticated animals and even humans [3, 4], thus attracting considerable attention. In this study, we attempt to search for potential vectors for BTV, EHDV, PALV and TIBOV from *Culicoides* collected from 16 counties in Yunnan Province, China. Generally, the areas in the south of the Ailao mountain chain are rainy, and the areas in the north of the Ailao mountain chain are dry (Fig. 1) [47]. Therefore, the midges collected from north Yunnan were usually too few to be investigated.

A total of 46 *Culicoides* species were recognized. A species close to *Culicoides nielamensis* Liu & Deng and belonging to *C.* subgenus *Culicoides* was as yet undescribed and temporarily named *C.* sp. (*Culicoides*) [47]. The *C.* sp. near *liui* with an access number of AEB2690 on the Barcode of Life Data (BOLD) System [51] was close to the typical *Culicoides liui* (BOLD: AEB2691) but had a few differences in morphology and genetic sequences [52, 53]. The *Culicoides* sp. near *obsoletus* Meigen was an unidentified species belonging to Obsoletus complex and distributed across some places in Yunnan [47, 54]. The species informally named *Culicoides newsteadi* (Asia) by us is widespread in Asia and is frequently recognized as *Culicoides punctatus* Latreille by Asian researchers [54–57]. This species is closer to *Culicoides newsteadi* Austen than *C. punctatus* in morphology, while its *cox1* sequence is different from *cox1* of both *C. newsteadi* and *C. punctatus* described in Europe [47]. *Culicoides sumatrae* Macfie is widespread in south Yunnan but is always incorrectly identified as other species such as *Culicoides nipponensis* Tokunaga [39, 56]. The principal Chinese classification manual for *Culicoides* [1] cannot identify *C. sumatrae* specimens. Actually, *C. nipponensis* is very rare in Yunnan according to our investigations. *Culicoides elbeli* Wirth & Hubert and *Culicoides menglaensis* Chu cannot be distinguished by morphology under an anatomical lens; the only morphological difference is that *C. menglaensis* has cibarial armature while *C. elbeli* does not [58].

There are four criteria argued by the WHO to confirm a vector for arbovirus [59, 60]: (i) recovery or detection of a virus from wild-caught specimens free from visible blood; (ii) demonstration of the ability to become infected by feeding on a viremic vertebrate host or on an artificial substitute; (iii) demonstration of the ability to transmit biologically via biting and (iv) accumulation of field evidence confirming the significant association of the infected arthropods with the appropriate vertebrate population in which disease or infection is occurring. Briefly, for midges, carrying arbovirus physiologically is a necessary but not sufficient requirement to be a vector.

During a complete cycle of the transmission for *Culicoides*-borne viruses, an infection occurs in the gut of the adult female *Culicoides* after exposure to a viremic blood meal, and then the progeny viruses released from the gut infect the salivary glands of *Culicoides*; finally, viruses released in salivary glands will invade the mammal host through the second biting by *Culicoides* [61]. BTV is detectable in the salivary glands of the *Culicoides* host as early as 5—7 days post-infection (dpi) because of the exposure to BTV in the gut through artificial feeding [61]. The more viral loads in a midge, the higher the probability of it being infectious. The viruses in a vector should experience at least two proliferations (i.e. in the gut and salivary glands) before attacking mammal hosts, and the second proliferation in salivary glands is necessary to infect mammals. Therefore, a strong positive result (approximately Cq < 25) in RT-qPCR detection was considered the criterion for a midge being infectious [62, 63]. However, some factors, such as the situations of midge collection and preservation, would affect the completeness of viruses in collected midges [47]. Therefore, the viral loads in field-collected and long-time saved midges might be underestimated according to the Cq values. Moreover, the threshold values used in a qPCR experiment directly determine the Cq values and are set automatically by default by the qPCR machine. Therefore, we set the threshold value as 0.015 all the time to make the Cq values comparable between different batches.

Seven pools of conspecific parous + gravid females without blood meals were obviously positive for viruses. Concretely, one pool of *C. shortti* (Cq = 24.6) and one pool of *C. orientalis* (Cq = 29.1) were positive for BTV, one pool of *C. oxystoma* was positive for PALV (Cq = 29.9), and four pools of *C. jacobsoni* were positive for TIBOV (Cq = 19.9–29.1). These results were confirmed by RT-PCR and subsequent electrophoresis. It is suggested that these field-collected midges were natural physiological carriers and potential vectors of associated arboviruses (BTV, PALV and TIBOV).

EHDV was found to be highly prevalent among ruminants in Yunnan [40, 64], while, unexpectedly, the pools of midges were all negative for EHDV. This may partly be caused by the narrow spectrum of the primers/probe for EHDV used in this study. Due to the lack of sufficiently conserved regions in the genomes of EHDV strains, it is impossible to design a set of primers/probes to match all the known EHDV strains published by the NCBI, so this set of primers/probe for EHDV was designed for a few EHDV strains in Yunnan and was originally based on a strain from Shizong County in eastern Yunnan [49].

*Culicoides shortti*, which is one of the four species in the Shortti group [2], is rare in north Yunnan and is a minor species in south Yunnan [38, 39, 47, 54], while it is common and feeds on cattle in Thailand [65, 66]. In this investigation, *C. shortti* was proved to be a potential BTV vector for the first time. Therefore, *C. shortti* may be one of the major potential BTV vectors in Thailand.

*Culicoides orientalis* was widespread in Yunnan and dominant in southwestern Yunnan, such as in Ruili County. It was considered a suspected vector of BTV in Southeast Asia before 1985, but Wirth and Dyce posited that this conclusion might be due to confusing *Culicoides wadai* Kitaoka with *C. orientalis* during species identification [67]. Recently, BTV was detected in field-collected *C. orientalis* in Thailand [68], supporting its role as a suspected vector of BTV. Quite a few *C. orientalis* specimens positive for BTV have now been found in southwestern Yunnan (i.e. Ruili, Mangshi and Yingjiang).

*Culicoides oxystoma* is a widespread species worldwide [46, 65, 69–71] and is common in China [38, 56]. It was thought to be a potential CHUV vector by viral isolation in Japan in 1985 [72]. Recently, it was reported as a natural carrier of BTV [39, 68]. It has now been proved to be a natural carrier of PALV using the RT-qPCR method in this investigation. However, no strong positive results (Cq ≤ 25) of RT-qPCR tests concerning *C. oxystoma* have been reported yet. Summarily, *C. oxystoma*, which is a dominant species in many areas, is a natural carrier for some arboviruses but may not satisfy the requirement to infect mammals because of the low viral load in them.

*Culicoides jacobsoni* is widespread in Southeast Asia and north Australia [38, 46, 73, 74] and has recently been reported as a potential vector of BTV and TIBOV in Yunnan [38, 75]. In this investigation, *C. jacobsoni* was proved to be a potential TIBOV vector a second time, and a pool of *C. jacobsoni* was weakly positive for BTV. It seems that *C. jacobsoni* is more able to transmit TIBOV than BTV according to the data from this investigation.

*Trithecoides* species (spp.) are common in Southeast Asia [38, 46]. *Culicoides palpifer* Das Gupta & Ghosh and *Culicoides parahumeralis* Wirth & Hubert are the most dominant *Trithecoides* spp. in Yunnan and are dominant in many southern areas of Yunnan [38, 47, 54]. Most *Trithecoides* spp. in Yunnan, including *C. palpifer*, *C. parahumeralis*, *Culicoides fordae* Lee, *C. laoensis*, *Culicoides rugulithecus* Wirth & Hubert, *Culicoides anophelis* Edwards, *Culicoides paraflavescens* Wirth & Hubert*, Culicoides malipoensis* Liu & Ren and *Culicoides paksongi* Howarth, have yellow scuta and sometimes similar wing patterns. As *Trithecoides* were considered non-vectors and it was time-consuming to sort them, *Trithecoides* complex with yellow scutum was placed in the same pools for RT-qPCR tests.

So far, we have not found any parous females in *Trithecoides* in any of the collections, according to Dyce’s criterion [76] for identifying parous female *Culicoides*. There are two possibilities: (i) parous *Trithecoides* are non-existent; (ii) Dyce’s criterion is not suitable for *Trithecoides*. If the former is true, the *Trithecoides* spp. should die just after laying eggs. Therefore, *Trithecoides* would not have the opportunity to transmit arboviruses to mammals, regardless of whether they can be infected by arboviruses. In line with this, only one case of arbovirus infection on *Trithecoides* species, in which *C. humeralis* was infected by AKAV artificially [57], has been reported to date. None of the *Trithecoides* spp. yielded a confidently positive result in our previous [38, 75] or current RT-qPCR detections.

This investigation suggested that BTV was widely circulated in the south of Yunnan, and the BTV-positive rate of conspecific pools even reached 50% at a sheep farm in Ruili (Fig. 4b), where > 90% of *Culicoides* were *C. orientalis*. However, no BT case was reported by the farm. Unexpectedly, the tested samples from the north of Tengchong County were all negative (Fig. 4b), while we were told by the local workstation orally that quite a few suspected BT cases in sheep occurred in October 2023 there. These samples contained 59 pools from collection TCSx and five pools from collection MG1 and were mainly *C. tainanus* followed by *Culicoides marginus* Chu and *Culicoides pastus* Kitaoka. As the collection TCSx was collected and then posted to Tengchong downtown by the farm host, we were not sure whether the work of collection was done correctly. Besides, the circulations of PALV and TIBOV were not so common compared with BTV. As for EHDV, the primers and probes for RT-qPCR should be developed, because the negative results of RT-qPCR in this study obviously did not match the serological investigation between 2014 and 2019 [40].

The pooling strategy used in this study had the advantage of testing many midges, but it had some limitations. First, species recognition was based on midge morphology only because Sanger sequencing would be unsuccessful if the target gene was heterogeneous for the pool of midges. Therefore, the strategy was only suitable for familiar species. Second, although ethanol was removed from the surface of the midges using absorbent paper before digestion, it was absorbed by midges; this might reduce proteinase activity and therefore the yield of nucleic acids. The concentration of residual ethanol in a pool of midges (20 midges/60 μl buffer = 0.33) was approximately 13-fold the concentration of ethanol in a tube with single midge (1 midge/40 μl buffer = 0.025). Additionally, if a pool of midges contained only one positive midge, the RT-qPCR read would be slightly underestimated compared with the results of a single-midge strategy because approximately 67% lysate (40 μl/60 μl) was obtained from pooled midges to purify nucleic acids compared to approximately 75% lysate (30 μl/40 μl) from a single midge.

## Conclusions

In this investigation, *C. shortti* was reported as a potential BTV vector for the first time. It was proven that *C. jacobsoni* was a potential TIBOV vector and *C. orientalis* was a potential BTV vector. *Culicoides oxystoma* was also proven to be a natural carrier of PALV using the RT-qPCR method. *Trithecoides* spp. might have a short lifespan, making them unlikely to be arbovirus vectors.

## Supplementary Information

Additional file 1.

Additional file 2.

Additional file 3.

Additional file 4.

## Data Availability

No datasets were generated or analysed during the current study.

## Abbreviations

AHSV: Africa horse sickness virus
AKAV: Akabane virus
BOLD: Barcode of Life data
BTV: Bluetongue virus
CHUV: Chuzan virus
Cq: Quantification cycle
dpi: Days post-infection
EEV: Encephalitis virus
EHDV: Epizootic hemorrhagic disease virus
NCBI: National Center for Biotechnology Information
NJ: Neighbor-joining method
NC: Negative control
PALV: Palyam virus
PCR: Polymerase chain reaction
RT-PCR: Reverse transcription-PCR
RT-qPCR: Reverse transcription-quantitative PCR
TIBOV: Tibet orbivirus
