# Supplementary material for: Searching for potential Culicoides vectors of four orbiviruses in Yunnan Province, China
Source: Parasit Vectors. 2025 Feb 24;18:73. doi: 10.1186/s13071-025-06679-1 (PMC11854118; doi:10.1186/s13071-025-06679-1)
Supplement: Supplementary file 2 — Additional file 2. [file 13071_2025_6679_MOESM2_ESM.docx]

**Table S1** The primers and probes used in this study

| Application | Target | Primer and probe sequence | Product length (bp) | Reference |
| --- | --- | --- | --- | --- |
| RT-qPCR | BTV (NS3) | F: 5’-TGGAYAAAGCGATGTCAAA | 97 | Hofmann *et al*. [48] |
|  |  | R: 5’-ACATCATCACGAAACGCTTC |  |  |
|  |  | P: 5’-FAM-ARGCTGCATTCGCATCGTACGC-BHQ1 |  |  |
|  | EHDV (NS1) | F: 5’-TCTTCGTCGACTGCCATCGAG | 83 | Yang *et al*. [49] |
|  |  | R: 5’-AACATTTTGACATGATTKGCRTARTAACT |  |  |
|  |  | P: 5’-VIC-TGGAGCGCTTTTTGAGAAAATACAACATGA-BHQ1 |  |  |
|  | PALV (VP7) | F: 5’-TACCYTATCTAGTGTGACTGATGC | 131 | Self-designed |
|  |  | R: 5’-TGAAGCCTGATTTYGATATGATTGG |  |  |
|  |  | P: 5’-Cy3-TCTGTACGCTTGCGCCACGC-BHQ2 |  |  |
|  | TIBOV (VP6) | F: 5’-CTACGGAACGAGGAGGGGAT | 100 | Yang *et al*. [50] |
|  |  | R: 5’-CTCGCTGCACATTTCCATCTC |  |  |
|  |  | P: 5’-FAM-ATCAGCTCGTCCTCCTCCCTCTCGT-BHQ1 |  |  |
| RT-PCR | BTV (Seg10) | BTV-2024-F: 5’-GTTAAAAAGTGTCGCTGCCA | 805 | Duan *et al*. [47] |
|  |  | BTV-2024-R: 5’-CGCACCCTCCCCCGTTATA |  |  |
|  | PALV (Seg7) | PALV-2024-F: 5’-ATGTGCGTAGATATGACAATAGC | 860 | Self-designed |
|  |  | PALV-2024-R: 5’-GCTCATCTGTACGCTTGC |  |  |
|  | TIBOV (Seg7) | TIBOV-2024-F: 5’-ATGGACGCAATCGCAGC | 1050 | Self-designed |
|  |  | TIBOV-2024-R: 5’-CTATTGGTACGCGGCAC |  |  |
