# Supplementary material for: Searching for potential Culicoides vectors of four orbiviruses in Yunnan Province, China
Source: Parasit Vectors. 2025 Feb 24;18:73. doi: 10.1186/s13071-025-06679-1 (PMC11854118; doi:10.1186/s13071-025-06679-1)
Supplement: Supplementary file 3 — Additional file 3. [file 13071_2025_6679_MOESM3_ESM.docx]

**Table S2** The dominant *Culicoides* species in the collections used

| Collection | |  | Dominant species and their percentages in collection | | | Amount **^a^** |
| --- | --- | --- | --- | --- | --- | --- |
| County | ID |  | Most dominant | Second most dominant | Third most dominant |  |
| Hekou | O1 |  | *C. palpifer* (68%) | *C. parahumeralis* (6%) | *C. orientalis* (5%) | 80,000 |
|  | O2 |  | *C. innoxius* (42%) | *C. palpifer* (25%) | *C. sumatrae* (9%) | 24,000 |
| Jiangcheng | M1 |  | *C. sumatrae* (34%) | *C. palpifer* (22%) | *C. jacobsoni* (21%) | 10,000 |
|  | M2 |  | *C. jacobsoni* (28%) | *C. sumatrae* (18%) | *C. palpifer* (11%) | 16,000 |
| Jinghong | EV21 |  | *C. orientalis* (73%) | *C. sumatrae* (4%) | *C. tainanus* (3%) | 5,000 |
| Lancang | V1 |  | *C. tainanus* (72%) | *C. rugulithecus* (9%) | *C. parahumeralis* (7%) | 4,000 |
| Lufeng | J1 |  | *C. arakawae* (77%) | *C. oxystoma* (15%) | *C. imicola* (5%) | 5,000 |
| Mangshi | MS |  | *C. orientalis* (68%) | *C. jacobsoni* (11%) | *C. oxystoma* (8%) | 33,000 |
| Menghai | X1 |  | *C. arakawae* (92%) | *C. oxystoma* (7%) | *C. guttifer* (0.4%) | 6,000 |
|  | X2 |  | *C. jacobsoni* (18%) | *C. tainanus* (15%) | *C. sumatrae* (15%) | 8,000 |
| Mengla | MLa21 |  | *C. innoxius* (20%) | *C. tainanus* (16%) | *C. sumatrae* (14%) | 150,000 |
| Puer | T2 |  | *C. jacobsoni* (49%) | *C. palpifer* (26%) | *C. sumatrae* (7%) | 4,000 |
| Ruili | R1 |  | *C. orientalis* (83%) | *C. sumatrae* (7%) | *C. oxystoma* (3%) | 75,000 |
| Tengchong | MG1**^b^** |  | NA | NA | NA | ≈2,000 |
|  | TCSx |  | *C. tainanus* (87%) | *C.* *marginus* (9%) | *C. pastus* (2%) | 450,000 |
|  | Q1 |  | *C. tainanus* (41%) | *C.* sp nr *obsoletus* (36%) | *C. newsteadi* (15%) | 9,000 |
|  | TC-1**^b^** |  | NA | NA | NA | ≈2,000 |
| Ximeng | W1 |  | *C. palpifer* (31%) | *C. jacobsoni* (17%) | *C.* sp nr *laoensis***^c^** (15%) | 48,000 |
| Yingjiang | S1 |  | *C. oxystoma* (67%) | *C. arakawae* (8%) | *C. parahumeralis* (7%) | 16,000 |
|  | S2 |  | *C. oxystoma* (56%) | *C. orientalis* (34%) | *C. tainanus* (3%) | 12,000 |
| Yuanjiang | N1 |  | *C. oxystoma* (89%) | *C. innoxius* (3%) | *C. imicola* (3%) | 30,000 |
| Yuanyang | K1 |  | *C. innoxius* (56%) | *C. imicola* (12%) | *C. orientalis* (10%) | 7,000 |
|  | K2 |  | *C. oxystoma* (52%) | *C. innoxius* (11%) | *C. jacobsoni* (10%) | 15,000 |
| Yulong | G1 |  | *C. tainanus* (99%) | *C. pastus* (0.3%) | *C. newsteadi* (0.2%) | 2,000 |

^a^ Estimated total number of *Culicoides* collected.

^b^ The midges from MG1 and TC1 were not counted and the residual midges were dropped after experiments. NA = not available.

^c^ An unidentified species similar to *C. laoensis* in morphology.
