# Supplementary material for: Searching for potential Culicoides vectors of four orbiviruses in Yunnan Province, China
Source: Parasit Vectors. 2025 Feb 24;18:73. doi: 10.1186/s13071-025-06679-1 (PMC11854118; doi:10.1186/s13071-025-06679-1)
Supplement: Supplementary file 4 — Additional file 4. [file 13071_2025_6679_MOESM4_ESM.docx]

**Table S3** Pools of conspecific midges detected by RT-qPCR

| Species | Status **^a^** | The number of tested | |  | Cq ≤ 30 | | |  | 30 < Cq ≤ 35 | | |  | Cq > 35 | | |
| --- | --- | --- | --- | --- | --- | --- | --- | --- | --- | --- | --- | --- | --- | --- | --- |
|  |  | pools | midges |  | BTV | PALV | TIBOV |  | BTV | PALV | TIBOV |  | BTV | PALV | TIBOV |
| *C. arakawae* | p | 19 | 274 |  | 0 | 0 | 0 |  | 0 | 0 | 0 |  | **1** | 0 | 0 |
| *C. homotomus* | p | 2 | 35 |  | 0 | 0 | 0 |  | 0 | 0 | 0 |  | 0 | 0 | 0 |
| *C. imicola* | p | 4 | 65 |  | 0 | 0 | 0 |  | 0 | 0 | 0 |  | 0 | 0 | 0 |
| *C. innoxius* | p | 28 | 402 |  | 0 | 0 | 0 |  | 0 | 0 | 0 |  | **1** | 0 | 0 |
| *C. insignipennis* | p | 1 | 5 |  | 0 | 0 | 0 |  | 0 | 0 | 0 |  | 0 | 0 | 0 |
| *C. jacobsoni* | p | 61 | 1154 |  | 0 | 0 | **4** |  | 0 | 0 | 0 |  | **1** | 0 | 0 |
| *C. laoensis* | n | 6 | 101 |  | 0 | 0 | 0 |  | 0 | 0 | 0 |  | 0 | 0 | 0 |
| *C. liui* | p | 3 | 47 |  | 0 | 0 | 0 |  | 0 | 0 | 0 |  | 0 | 0 | 0 |
| *C. marginus* | p | 3 | 61 |  | 0 | 0 | 0 |  | 0 | 0 | 0 |  | 0 | 0 | 0 |
| *C. marginus* | bf | 3 | 66 |  | 0 | 0 | 0 |  | 0 | 0 | 0 |  | 0 | 0 | 0 |
| *C. newsteadi* (Asia) | bf | 20 | 300 |  | 0 | 0 | 0 |  | **2** | 0 | 0 |  | **1** | 0 | 0 |
| *C.* sp nr *obsoletus* | p | 10 | 190 |  | 0 | 0 | 0 |  | 0 | 0 | 0 |  | 0 | 0 | 0 |
| *C. orientalis* | p | 75 | 1428 |  | **1** | 0 | 0 |  | **8** | 0 | 0 |  | **10** | 0 | 0 |
| *C. oxystoma* | p | 45 | 887 |  | 0 | **1** | 0 |  | 0 | 0 | 0 |  | **5** | **2** | 0 |
| *C. palpifer* | n | 6 | 125 |  | 0 | 0 | 0 |  | 0 | 0 | 0 |  | 0 | 0 | 0 |
| *C. pastus* | p | 7 | 101 |  | 0 | 0 | 0 |  | 0 | 0 | 0 |  | 0 | 0 | 0 |
| *C. shortti* | p | 2 | 47 |  | **1** | 0 | 0 |  | 0 | 0 | 0 |  | 0 | 0 | 0 |
| *C. spiculae* | p | 4 | 53 |  | 0 | 0 | 0 |  | 0 | 0 | 0 |  | 0 | 0 | 0 |
| *C. sumatrae* | p | 42 | 608 |  | 0 | 0 | 0 |  | 0 | 0 | 0 |  | **5** | 0 | 0 |
| *C. tainanus* | p | 105 | 2035 |  | 0 | 0 | 0 |  | **2** | 0 | 0 |  | 0 | 0 | 0 |
| *C. tainanus* | bf | 17 | 335 |  | 0 | 0 | 0 |  | **1** | 0 | 0 |  | **2** | 0 | 0 |
| *Trithecoides* **^b^** | n | 80 | 1576 |  | 0 | 0 | 0 |  | 0 | 0 | 0 |  | 0 | 0 | 0 |
| **Total** |  | **543** | **9895** |  | **2** | **1** | **4** |  | **13** | **0** | **0** |  | **26** | **2** | **0** |

**^a^** The midge status included bf = blood fed female, p = parous female without blood meal, and n = nulliparous female without blood meal.

**^b^** That meant *C.* subgenus *Trithecoides* complex with yellow scutum, which mainly included *C. palpifer* and *C. parahumeralis*, and included a few of *C. laoensis*, *C. rugulithecus*, *C. malipoensis*, *C. paraflavescens*, *C.* *paksongi*, and so on.
